# Auswirkungen des COVID-19-Lockdowns auf physische Leistungsparameter im professionellen Fußball: Eine narrative Literaturübersicht

**DOI:** 10.1007/s40664-022-00455-z

**Published:** 2022-01-24

**Authors:** D. Friebe, M. Fischer, F. Giesche, E. Füzéki, W. Banzer

**Affiliations:** Institut für Arbeits‑, Sozial- und Umweltmedizin, Arbeitsbereich Präventiv- und Sportmedizin, Goethe-Universität Frankfurt/Main, Theodor-Stern-Kai 7, Haus 9B, 60590 Frankfurt/Main, Deutschland

**Keywords:** Coronavirus, Detraining, Professioneller Fußball, Leistungsfähigkeit, Heimtraining, Coronavirus, Detraining, Elite football, Performance, Hometraining

## Abstract

**Hintergrund:**

Die staatlichen Maßnahmen zur Eindämmung des Coronavirus SARS-CoV‑2 im Jahr 2020 brachten den Trainings- und Wettkampfbetrieb im professionellen Fußball in vielen Ländern zum zeitweiligen Erliegen. In Folge des Lockdowns waren die Trainingsmöglichkeiten zumeist auf unspezifische heimbasierte Trainingsmethoden begrenzt. Es ist unklar, ob sich die fehlenden sportspezifischen Belastungsreize negativ auf die physische Leistungsfähigkeit der Fußballspielenden auswirkten.

**Methodik:**

Im Rahmen eines narrativen Reviews wurde mittels einer selektiven Literaturrecherche in den Datenbanken PubMed, Google Scholar und BISp-Surf nach Studien gesucht, welche die Auswirkungen des Lockdowns auf physische Leistungsparameter bei erwachsenen professionellen Fußballspielenden untersuchten.

**Ergebnisse:**

In die Übersichtsarbeit wurden sechs prospektive Längsschnittstudien eingeschlossen. In allen Studien kam während der Quarantäne ein heimbasiertes Ersatztraining zum Einsatz. Vier Studien verglichen die Leistungsfähigkeit der Fußballer/-innen mit Leistungsdaten aus vorherigen Spielzeiten. Zwei Studien ermittelten die Leistungsfähigkeit der Sportler/-innen unmittelbar vor und nach der Lockdownperiode.

**Diskussion:**

Während die allgemeine Kraft- und Ausdauerleistung durch heimbasierte Ersatztrainingsprogramme erhalten werden kann, weisen die Studien darauf hin, dass sich die fehlenden spezifischen Belastungsreize vor allem negativ auf die Schnelligkeits- und Schnellkraftleistung der Fußballspielenden auswirken könnten. Bei Rückkehr in den regulären Trainingsbetrieb sollte daher auf eine progressive Belastungssteuerung insbesondere im Schnelligkeitstraining geachtet werden, um das Risiko für Verletzungen zu senken.

## Abstract (en)

**Background:**

Governmental measures in 2020 to contain the severe acute respiratory syndrome coronavirus type 2 (SARS-CoV-2) brought training and competition in professional soccer to a temporary halt in many countries. As a result of the lockdown, training possibilities were mostly limited to nonspecific home-based training methods. It is unclear whether the lack of sport-specific stimuli led to a reduction in the physical performance of soccer players.

**Methods:**

For the narrative review, the PubMed, Google Scholar and BISp-Surf databases were selectively searched for studies examining the effects of the lockdown on physical performance parameters in adult professional soccer players.

**Results:**

In this review six prospective longitudinal studies were included. In all studies home-based training was performed during quarantine. Of the studies four compared the performance of the soccer players with data from previous seasons and two studies assessed the performance of players immediately before and after the lockdown period.

**Discussion:**

While general strength and endurance performances can be maintained through home-based substitute training programs, the studies indicated that the lack of football-specific stimuli could have an impact on the rapid power and speed performances of soccer players. Progressive loading control especially for speed training should be considered when returning to regular training in order to reduce the risk of injuries.

Der erste COVID-19-Lockdown führte im Jahr 2020 zu erheblichen Einschränkungen des Trainings- und Wettkampfbetriebes im professionellen Fußball. Die Fußballspielenden hatten in Folge von häuslichen Quarantänen oder Ausgangsbeschränkungen häufig keine oder nur eingeschränkte Trainingsmöglichkeiten. Die Auswirkungen dieser Phase mit fehlenden sportartspezifischen Belastungsreizen auf die körperliche Leistungsfähigkeit wurden bislang wenig erforscht. Die vorliegende narrative Übersichtsarbeit fasst die Befundlage hierzu zusammen. Die gewonnenen Erkenntnisse liefern wichtige Ansätze für eine optimierte Trainingsgestaltung und Verletzungsprävention nach Wiederaufnahme des regulären Trainings- und Wettkampfbetriebes.

Die hochinfektiöse Coronaviruserkrankung 2019 (COVID-19) hat sich im Frühjahr 2020 zu einer weltweiten Pandemie entwickelt [23]. Da zu diesem Zeitpunkt weder ein wirksamer präventiver Impfstoff noch geeignete Pharmazeutika zur Verfügung standen, kamen in vielen Ländern Maßnahmen des öffentlichen Gesundheitswesens (Hygieneregeln, Kontakteinschränkungen, soziale Distanzierung, Isolation und Quarantäne) zum Einsatz, um die Ausbreitung des Virus einzudämmen [15]. Im Breiten- und Freizeitsport führten die staatlich verordneten Maßnahmen zur Schließung von Vereins- und Trainingsstätten [11, 18]. Im professionellen Fußball kam es in Folge zu Verschiebungen, Unterbrechungen und teilweise sogar zu vollständigen Ausfällen von nationalen und internationalen Spielen [2]. Durch die räumlichen Einschränkungen war auch ein strukturiertes und sportspezifisches Training zeitweise nur bedingt oder gar nicht möglich. Als Alternative führten die Fußballspielenden in der Regel Heimtrainingsprogramme durch, welche sich zumeist aus Kraft- und Ausdauermethoden zusammensetzten. Die fehlenden oder eingeschränkten sportartspezifischen trainings- und wettkampfassoziierten Belastungsreize im Heimtraining könnten zu negativen physiologischen Anpassungen, sog. Detraining-Effekten [4, 14], der Sporttreibenden führen. Bei der Rückkehr in den regulären Trainings- und Wettkampfbetrieb könnten diese Detraining-Anpassungen die Spielenden einem erhöhten Verletzungsrisiko aussetzen [4]. Hinweise darauf liefern u. a. Daten aus der National Football League. Dort kam es im Jahr 2011 streikbedingt (Lockout) über mehrere Monate zu Abweichungen des regulären Trainings- und Spielbetriebs. In dieser Zeit wurde ein Anstieg der Inzidenz von Achillessehnenverletzungen beobachtet [14].

Die Auswirkungen des COVID-19-Lockdowns auf die körperliche Leistungsfähigkeit von professionellen Fußballspielenden sind bislang noch wenig erforscht. Gemäß den Prognosen mathematischer Modellierungsstudien könnte es im Zusammenhang mit dem Coronavirus bis 2022 jedoch zu erneuten zeitweiligen Einschränkungen des sozialen und öffentlichen Lebens kommen [12]. Eine strukturierte Zusammenstellung der bisherigen Befundlage zu den Auswirkungen derartiger Lockdownperioden auf fußballspezifische Leistungsparameter könnten daher wichtige Potenziale für die inhaltliche Trainings- und Belastungssteuerung während oder nach einem erneuten Lockdown bieten und den Fußballspielenden eine möglichst sichere Wiederaufnahme ihrer Spiel- und Wettkampfaktivität ermöglichen [17].

## Methodik

Im Rahmen dieser narrativen Übersichtsarbeit wurden mittels selektiver Literaturrecherche Studien zu den Auswirkungen des ersten COVID-19-Lockdowns im Jahr 2020 auf physische Leistungsparameter im professionellen Fußball identifiziert. Die Recherche wurde in den Datenbanken MEDLINE (PubMed), Google Scholar und BISp-Surf (Bundesinstitut für Sportwissenschaften) durchgeführt. Mittels eines systematischen Suchterms (covid-19 OR cov-19 AND Lockdown OR Quarantine AND professional OR Elite AND football OR Soccer) wurden bis Dezember 2021 publizierte Studien recherchiert. Darüber hinaus wurden die Referenzlisten der eingeschlossenen Studien durchsucht, um weitere potenziell geeignete Publikationen zu identifizieren. In das vorliegende Review wurden prospektive Längsschnittstudien eingeschlossen, welche die Auswirkungen der ersten Coronoavirus-bedingten Lockdownperiode auf körperliche Leistungsparameter professioneller, erwachsener Fußballspielender nach der Lockdownperiode mit vorangegangenen Zeitpunkten verglichen. Untersuchungen, welche die Leistungsparameter (z. B. Laufleistung) lediglich innerhalb der Spiele erfassten, wurden nicht berücksichtigt. Folgende Daten wurden extrahiert: Stichprobencharakteristik, Dauer und Methoden der Trainingsintervention während des Lockdowns, Studiendesign und statistische Analysen, Messgrößen und Resultate. Parameter der Ausdauer‑, Kraft‑, Schnelligkeits- und Schnellkraftleistung stellten die Hauptzielgrößen dar.

Aufgrund des narrativen Charakters der vorliegenden Arbeit wurde keine systematische Bewertung der methodischen Qualität der eingeschlossenen Studien durchgeführt.

## Ergebnisse

In die narrative Übersichtsarbeit wurden 6 Studien eingeschlossen. Vier Studien [6, 7, 10, 20] verglichen die Leistungsfähigkeit der Fußballspielenden mit Leistungsdaten aus vorherigen Spielzeiten. Zwei Studien [1, 16] ermittelten die Leistungsfähigkeit der Sportler/-innen unmittelbar vor und nach der Lockdownperiode. Die Maßnahmen zur Eindämmung des Coronavirus variierten zwischen den Ländern und Regionen der einzelnen Studien und werden daher bei Erstnennung kurz erläutert. Tab. 1 gibt einen detaillierten Überblick über die Studiencharakteristik und die Resultate der einzelnen Studien.

**Table 1:** 

Studie	Stichprobe	Training während Quarantäne	Studiendesign/Statistik	Outcomes (Einheiten; signifikante relevante Differenzen Prä- vs. Post-Lockdown)
Albuquerque Freire et al. (2020) [1]	*20 professionelle Fußballspieler der brasilianischen Liga, Serie A*	*Dauer: 6 Wochen*	**Design:**	**Messwerte der Laufleistung:**
			*Prä- vs. Post-COVID-19-Quarantäne*	
	*Alter: 26* *±* *4 Jahre; Größe: 179* *±* *6* *cm; Gewicht: 76,9* *±* *7* *kg*	*Häufigkeit: 3‑mal/Woche*	**Datenerhebung:**	Totale Distanz (m)
		**Heimtraining:**	Yo-Yo-Intermittent-Ausdauer-Test mit 5‑Hz GPS-Einheit (20 m Shuttle-Lauf; Anfangsgeschwindigkeit: 10 Km/h, Final: 19 km/h, Pause zwischen den Shuttle-Läufen: 5 s)	Relative Distanz (m/min; **−12,5** **%**)
		10 min Warm-up/Cool-down	**Statistik:**	Sprints (Frequenz)
		15 min Mini-Band-Workout (z. B. Sprünge, Ausfallschritte, Burpees etc.)	*t‑Test für verbundene Stichproben/Wilcoxon-Test*	Max. Geschwindigkeit (km/h; **−2,9** **%**)
		15 min funktionelle Übungen (z. B. Mobilitätsübungen, Skippings in unterschiedlichen Varianten, Koordination mit Ball, hochintensive Sprints)		Beschleunigungen > 2 m/s^2^ (Frequenz; **−13,3** **%**)
		15 min Fahrrad-Workout		Abbremsfrequenz > 2 m/s^2^ (Frequenz; **−19,8** **%**)
				Richtungswechsel
				Explosive Bewegungen (Frequenz)
				Hochintensive Laufdistanz (m)
				Distanz (m) mit geringerer (< 11 km/h) moderater (> 11 < 15,5 km/h) und hoher Intensität (15,5 > 19 km/h)
				Gesamtzeit des Tests (min)
				Max. Hf (bpm)
				VO_2max_ (ml/kg/min)
Rampinini et al. (2021) [20]^a^	*50 professionelle Fußballspieler der italienischen Serie A*	*Dauer: 13 Wochen*	**Design:**	**Messwert der Laufleistung:**
	*Alter: 25,4* *±* *5 Jahre; Größe: 182* *±* *5* *cm; Gewicht: 78,5* *±* *5,7* *kg*	*Häufigkeit: 8‑mal/Woche*	*Prä- vs. Post-COVID-19-Quarantäne im Vergleich zu 3 weiteren Prä-Post-Perioden der vergangenen 5 Jahre*	Laktatkonzentration (mmol/l; **signifikant reduziert**)
		**Heimtraining**:	**Datenerhebung:**	**Messwerte der Sprungleistung:**
		4–5 Ausdauereinheiten/Woche (mittlere bis hohe Intensität) (Laufband oder Fahrrad)	Mognoni-Test: 6‑min-Lauf mit konstanter Geschwindigkeit: 13,5 km/h, 1350 m, Geschwindigkeit über akustisches Signal aufrechterhalten	Sprunghöhe (cm)
		2–3 Krafttrainingseinheiten pro Woche (eigenes Körpergewicht oder leichte Zusatzgewichte)	Contermovement-Jumps auf Kraftmessplatte	Absoluter und relativer Peak-Power-Output (W; als Korrelat der Beinkraftleistung; **signifikant reduziert**)
			**Statistik:**	
			*Lineare gemischte Modelle zur Prüfung von Veränderungen innerhalb und zwischen den Perioden (COVID-19 vs. 3 weitere Perioden)*	
Grazioli et al. (2020) [10]	*23 professionelle Fußballspieler der brasilianischen Liga, Serie A*	*Dauer: 9 Wochen*	**Design:**	**Messwert der Laufleistung:**
	*Alter: 26,3* *±* *5,6 Jahre; Gewicht: 78,4* *±* *8,7* *kg*	*Häufigkeit: keine Angaben*	*Prä- vs. Post-COVID-19-Quarantäne im Vergleich zu einer weiteren Prä-Post-Periode der Saisonpause 2019*	*Gesamtlaufdistanz (m)*
		**Heimtraining:**	**Datenerhebung:**	**Messwert der Sprungleistung:**
		Warm-up/Mobilitätsübungen der unteren Extremität	Yo-Yo-Intermittent-Ausdauer-Test (4 × 15 m Shuttle-Lauf; Anfangsgeschwindigkeit: 9 km/h, Steigerung 1 km/h, Pause zwischen den Shuttle-Läufen: 10 s)	*Sprunghöhe CMJ (cm; *−**3,5** **%***)*
		Funktionelle Übungen mit dem eigenen Körpergewicht (z. B. 3 × 10–15 Wiederholungen von Sprüngen, Ausfallschritten, seitliche Kniebeugen, Hamstring-Übungen etc.)	Squat Jumps und Counter Movement Jumps auf einer Kontaktplattform	*Sprunghöhe SJ (cm)*
			Nordic-Hamstring-Curl mit Kraftaufnahme (exzentrische Kniebeuger)	**Messwerte der Kraftleistung:**
			Sprint mittels Lichtschrankenmessung bei Start, 10 m und 20 m	Absolute Kraft (N)
			**Statistik:**	Relative Kraft (N/kg)
			*t‑Test für verbundene Stichproben, Effektstärke*	**Messwert der Sprintleistung:**
				10 m Sprintzeit (s; **6,9** **%**)
				20 m Sprintzeit (s; **4,3** **%**)
Pedersen et al. (2021) [16]	*8 professionelle Fußballspielerinnen (Norwegen, Level 3)*	*Dauer: 12 Wochen*	**Design:**	**Messwert der Kraftleistung:**
	*Alter: 18,8* *±* *1,9 Jahre; Größe 168* *±* *4* *cm; Gewicht 61,3* *±* *3,7* *kg*	*Wöchentliche Trainingszeit: 233* *±* *47* *min*	*Prä- vs. Post-COVID-19*	*Einerwiederholungsmaximum (kg)*
		**Heimtraining:**	*4 Wochen Heimtraining*	**Messwerte der Sprungleistung:**
		Individuelles und kontaktloses Gruppentraining bestehend aus Kraft‑, Schnelligkeit‑, Sprung- und Laufübungen	*8 Wochen kontaktloses Kleingruppentraining ohne Equipment*	Sprunghöhe (cm)
			**Datenerhebung:**	Absprunggeschwindigkeit (ms)
			Kniebeugen	Maximale und mittlere Kraft (N)
			Contermovement-Jumps auf Kraftmessplatte	Maximale und mittlere Leistung (W)
			Sprint mittels Lichtschrankenmessung bei Start, 5 m, 10 m und 15 m	Flugzeit (ms)
			**Statistik:**	**Messwert der Sprintleistung:**
			*t‑Test für verbundene Stichproben*	5 m, 10 m & 15 m Sprintzeit (s)
Cohen et al. (2020) [7]	*16 professionelle Fußballspieler der 1. kolumbianischen Liga*	*Dauer: 15 Wochen*	**Design:**	**Messwerte der Sprungleistung:**
	*Alter: 24,3* *±* *3,8 Jahre; Größe: 179* *±* *7* *cm; Gewicht: 79,2* *±* *9* *kg*	*Häufigkeit: 5‑ bis 6‑mal/Woche*	*Prä- vs. Post-COVID-19-Quarantäne im Vergleich zu einer weiteren Prä-Post-Periode der Saisonpause 2019 (12 Spieler)*	*Sprunghöhe (cm)*
		**Heimtraining:**	**Datenerhebung:**	*Reaktivkraftindex (Sprunghöhe/Kontraktionszeit)*
		Team-Zirkeltraining, digital ab 20. März (5-mal/Woche)	Counter Movement Jumps auf Kraftmessplatte	**Exzentrische Phase:**
		April bis Juni: individuelle Trainingspläne: Kraftzirkel mit Körpergewicht und elastischen Bändern (5-mal/Woche)	**Statistik:**	Kraftentwicklungsrate (zw. Bewegungsinitiierung und Beginn der Abbremsung/Verlangsamung neg. Beschleunigung; Exzentrik) (N/s/kg; **−17,3** **%**) und Dauer (s; **15,7** **%**)
		Juni: Kraftzirkel (6-mal/Woche) + intermittierendes Intervalltraining mit und ohne Ball (2-mal/Woche)	*t‑Test für verbundene Stichproben, Cohen d Effektstärken*	Kraft bei 0‑Geschwindigkeit (zwischen Abbremsung/Verlangsamung der neg. Beschleunigung; Exzentrik/Beginn Konzentrik; N/kg)
				Kraftentwicklungsrate (zw. Beginn der Abbremsung/Verlangsamung neg. Beschleunigung und tiefster Punkt der exzentrischen Phase) (N/s/kg)
				Maximalgeschwindigkeit der neg. Beschleunigung und Leistung der exzentrischen Phase (m/s)
				**Konzentrische Phase:**
				Spitzengeschwindigkeit pos. Beschleunigung (s)
				Spitzenkraft (N/kg)
				Kraft bei Spitzenleistung (N/Kg; **−3,4** **%**)
				Leistung zum Absprung (W/kg)
				Dauer der konzentrischen Phase (s)
				*Landephase*
				Maximale Bodenreaktionskraft (N/Kg, **−13,1** **%**)
				Kraftentwicklungs‑/Belastungsrate (N/s/kg, **−26,5** **%**)
Cavarretta et al. (2021) [6]	*29 professionelle Fußballspieler der italienischen Serie A*	*Dauer: 7 Wochen*	**Design:**	**Messwerte der Ergometerleistung:**
	*Alter: 27 Jahre (23;31); Gewicht: 81* *kg (77; 86)*	*Häufigkeit: 34 Heimtrainingseinheiten*	*Prä- vs. Post-COVID-19 Quarantäne*	Maximalleistung (Watt)
		*2‑mal/Woche Übungen und Sprünge zur Kräftigung der unteren Extremität*	**Datenerhebung:**	Relative Leistung (Watt/kg)
		**Heimtraining:**	EKG-Ausbelastungstest auf dem Fahrradergometer (Protokoll: 50 W Inkrement alle 2 min)	Max. HF (bpm)
		19/34 Einheiten hochintensives Intervalltraining auf dem Radergometer (Woche 1–5)	**Statistik:**	
		15/34 Einheiten funktionelle Übungen/Rumpfstabilität	*Wilcoxon-Vorzeichen-Rang-Test*	
		10–50 m Sprintübungen außerhalb (Woche 6–7)		

*GPS* Global Positioning System, *Hf* Herzfrequenz, *VO2* Sauerstoffaufnahme, *W* Watt, *CMJ* Counter Movement Jump, *SJ* Squat Jump, *N* Newton

^a^Studien ohne numerische Angabe deskriptiver Ergebnisse

### Ausdauerleistung

Es konnten 4 Untersuchungen [1, 6, 10, 20] identifiziert werden, welche die Effekte des Lockdowns auf die Ausdauerleistung bei professionellen Fußballspielenden untersuchten.

Albuquerque Freire et al. (2020) [1] erfassten die Ausdauerleistungsfähigkeit von 20 männlichen professionellen Fußballspielern der höchsten brasilianischen Spielklasse mittels YO-YO-Intermittent-Ausdauer-Test vor und nach der 40-tägigen häuslichen Quarantäne. In dieser Phase führten die Spieler drei 30-minütige Zirkel- und Ausdauertrainingseinheiten pro Woche bei 65–75 % der maximalen Herzfrequenz durch. Hinsichtlich der VO_2max_ sowie der Laufdistanzen mit geringer und moderater Intensität waren keine quarantänebedingten Veränderungen zu beobachten (*p* > 0,05).

Im Rahmen der Studie von Rampinini et al. (2021) [20] wurde die aerobe Leistungsfähigkeit von 50 professionellen Fußballspielern der italienischen Serie A innerhalb von 4 Perioden (Prä‑/Post-COVID-19-Quarantäne 2020, Prä‑/Post-Sommerpause Spielzeit 2017) verglichen. Während des Lockdowns befanden sich die Spieler in Heimisolation und führten ein aerobes Laufband-Training mit moderater bis hoher Intensität an 4 Wochentagen sowie ein ergänzendes Krafttraining an 2 Tagen durch. Im Rahmen des verwendeten Mognoni-Tests konnte eine verbesserte aerobe Ausdauerleistung in Folge des Lockdowns festgestellt werden, welche sich in einer reduzierten Laktatkonzentration widerspiegelte.

Auch in der Untersuchung von Grazioli et al. (2020) [10] führte die 2‑monatige häusliche Quarantäne mit eingeschränkten Trainingsmöglichkeiten (heimbasiertes Zirkeltraining) bei 23 männlichen brasilianischen Fußballspielern zu keiner Reduzierung der kardiorespiratorischen Leistung im Vergleich zur vorausgegangenen regulären Saisonpause (23 Tage). Innerhalb des durchgeführten YO-YO-Tests konnten keine Unterschiede in der Ausdauerleistung zwischen den 2 Messzeitpunkten festgestellt werden (*p* > 0,05).

In Folge der Lockdownperiode in Italien untersuchten Cavarretta et al. (2021) [6] die kardiovaskuläre Leistungsfähigkeit von 29 professionellen männlichen Fußballspielern der Serie A innerhalb eines Belastungs-Stufentests auf dem Fahrradergometer. Mit dem Ziel des Erhalts der Leistungsfähigkeit führten die Spieler während der 7‑wöchigen häuslichen Quarantäne 34 Einheiten mit intensivem Ergometertraining, Kraft- und Stabilisationsübungen sowie Sprüngen durch. Weiterhin konnte in den letzten 2 Wochen durch Lockerungen der Lockdownmaßnahmen ein ergänzendes außerhäusliches Sprinttraining durchgeführt werden. In der Gegenüberstellung zur vorangegangenen regulären 2‑monatigen Saisonpause konnten keine signifikanten Veränderungen der maximalen Ergometerleistung oder Herzfrequenz detektiert werden.

### Kraftleistung

Zwei der eingeschlossenen Untersuchungen [10, 16] erfassten die Kraftleistung der Fußballspielenden im Zuge des Lockdowns mittels des Einwiederholungsmaximums der Kniebeuge bzw. der exzentrischen Kraft im Nordic-Hamstring-Curl.

Bei 23 männlichen Fußballern zeigten sich in Folge der 2‑monatigen häuslichen Quarantäne im Vergleich zur regulären Saisonpause keine Veränderungen in der relativen exzentrischen Kraftleistung der kniebeugenden Muskulatur innerhalb des Nordic-Hamstring-Curls (*p* > 0,05; [10]).

Pedersen et al. [16] untersuchten die Effekte eines 12-wöchigen heim- und kleingruppenbasierten (nach Lockerungen der staatlichen Maßnahmen) Trainings während des COVID-19-Lockdowns bei 8 norwegischen Fußballspielerinnen. Die Intervention beinhaltete 5 Wochen individuelle heimbasierte Kraft‑, Schnelligkeit‑, Sprung und Laufübungen, gefolgt von 7 Wochen kontaktlosem Kleingruppentraining. Hierbei konnte keine Reduktion der Kraftleistung in Folge des Lockdowns festgestellt werden (*p* = 0,28). Die Maximalkraftleistung in der Kniebeuge konnte über die spielfreie Lockdownperiode durch das organisierte heimbasierte Training erhalten werden.

### Schnelligkeits‑/Schnellkraftleistung

Fünf Studien [1, 7, 10, 16, 20] untersuchten den Effekt des Lockdowns auf Parameter der Schnelligkeits- bzw. Schnellkraftleistung.

Im Rahmen der Untersuchung von Albuquerque Freire et al. [1] zeigten die Spieler beim YOYO-Test eine signifikante Reduktion der maximalen Laufgeschwindigkeit (*p* = 0,021), der durchschnittlichen Laufgeschwindigkeit (*p* = 0,019), der Anzahl an Beschleunigungen (*p* = 0,03) sowie der Abbremsmanöver (*p* = 0,05).

Weiterhin konnten Rampinini et al. [20] eine Reduktion der Leistung im Counter Movement Jump feststellen. Verglichen mit der regulären Saisonpause, führte die COVID-19-bedingte Quarantäne bei den 15 Fußballspielern zu einem signifikant geringeren absoluten und relativen maximalen Power-Output (*p* < 0,001). Hinsichtlich der Sprunghöhe konnte kein Unterschied festgestellt werden (*p* > 0,05).

Auch in der Untersuchung von Grazioli et al. [10] führte die Lockdownperiode verglichen mit der regulären Saisonpause zu einer signifikanten Leistungsreduktion innerhalb mehrerer Schnelligkeitsparameter bei 23 männlichen Fußballspielern. Im Anschluss an die häusliche Quarantäne zeigte sich eine reduzierte Leistungsfähigkeit innerhalb der 10- und 20-m-Sprints (*p* < 0,001) sowie des Counter Movement Jumps (*p* = 0,006). Hinsichtlich des Squat Jumps zeigten sich keine Differenzen zwischen den Messzeitpunkten (*p* > 0,05).

Cohen et al. [7] erhoben bei 16 professionellen männlichen Fußballspielern eines kolumbianischen Erstligavereins neuromuskuläre, kinetische und temporale Parameter während der exzentrischen (Abwärtsbewegung) und konzentrischen (Aufwärtsbewegung) Phase von Counter Movement Jumps und deren Landung auf einer Kraftmessplatte vor und nach eines 15-wöchigen COVID-19-Lockdowns. Dabei führten die Spieler ein insgesamt 12 Wochen digital angeleitetes heimbasiertes Zirkeltraining und individuelles Krafttraining mit dem Körpergewicht und Widerstandsbändern durch. In den letzten 3 Wochen des Lockdowns sind zusätzlich wöchentlich 2 Einheiten intermittierendes Ausdauertraining im Freien absolviert worden. Zum Zeitpunkt nach dem COVID-19-Lockdown beobachtete die Autorengruppe eine signifikante Abnahme der exzentrischen Kraftentwicklungsrate (zwischen Bewegungsinitiierung und Beginn der Verlangsamung der negativen Beschleunigung; Yielding-Phase; *p* = 0,01) sowie eine signifikant längere exzentrische Phasendauer (*p* = 0,01) der Sprungbewegung. Die Kraft während der konzentrischen Spitzenleistung (*p* = 0,01) war signifikant reduziert. Die maximale Bodenreaktionskraft (*p* = 0,02) sowie die Kraftentwicklung- bzw. Belastungsrate (*p* = 0,04) während der Landung waren signifikant verringert.

Konträr zu den Ergebnissen der übrigen Studien wiesen die Profifußballerinnen in der Untersuchung von Pedersen et al. [16] keine Veränderung der Counter Movement Jump-Höhe (*p* = 0,09) und der 15-m-Sprintzeit (*p* = 0,52) in Folge der Lockdownperiode auf.

## Diskussion

Die vorliegende Übersichtsarbeit untersuchte die Auswirkungen des ersten COVID-19-Lockdowns auf zentrale physische Leistungsdeterminanten (Ausdauer‑, Kraft- sowie Schnelligkeitsfähigkeit [22]) im Fußball bei professionellen, erwachsenen Spieler/innen. Den Resultaten zufolge scheint sich ein fußballunspezifischeres Ersatztraining während des COVID-19-Lockdowns weniger stark auf die Ausdauer- und allgemeine Kraftleistungsfähigkeit als auf die Schnelligkeits- bzw. Schnellkraftleistung auszuwirken.

Die Ausdauer- und Kraftleistung konnte über die Lockdownperiode weitestgehend aufrechterhalten oder teilweise sogar verbessert werden. Potenziellen Detraining-Effekten der Ausdauerleistungsfähigkeit, die bereits nach 3 Wochen auftreten können [5, 8], scheint demnach mittels fußballunspezifischer heimbasierter Trainingsprogramme mit moderaten Intensitäten (z. B. Ergometer- oder Laufbandeinheiten; [1, 6, 20]) sowie zyklischen Ganzkörperübungen ([1, 6]; z. B. Jumping-Jacks, Burpees, Sprünge) während der Lockdownperiode entgegengewirkt werden zu können.

Ähnliches zeigt sich auch in Bezug auf die allgemeine Kraftfähigkeit von trainierten Sportler/-innen, welche gemäß Literatur über einen ähnlich langen trainingsfreien Zeitraum erhalten werden kann [13]. Erst nach einem Zeitfenster von ca. 3 Wochen scheinen negative Adaptionen hinsichtlich Muskelmasse, -struktur und -kraft aufzutreten. Zwar erstreckten sich die staatlichen Maßnahmen zur Reduzierung des COVID-19-Infektionsgeschehens in vielen Ländern über diesen Zeitraum hinaus, jedoch scheinen die angewandten heimbasierten Übungen mit eigenem Körpergewicht und Widerstandsbändern ausreichend, um die Kraftfähigkeit der professionellen Fußballspielenden zu erhalten [10, 16].

Im Gegensatz dazu zeigten die meisten Studien eine herabgesetzte Schnelligkeitsleistungsfähigkeit der Fußballspielenden in Folge der Lockdownperiode während Sprint- und Sprungtests [1, 20]. Durch die räumlichen und organisatorischen Einschränkungen im Rahmen der staatlich angeordneten COVID-19-Quarantäne in vielen südamerikanischen Ländern [1, 11] sowie in Italien [20] bestand das Training in diesen 3 Untersuchungen lediglich aus sportartunspezifischen heimbasierten Ausdauer- und Krafteinheiten. Übungen mit maximaler neuromuskulärer Ansteuerung, wie sie in Fußballspielen etwa in Form von Sprints, Sprüngen oder Schüssen vorkommen, waren in dieser Zeit nur sehr eingeschränkt oder gar nicht möglich. Frühere Untersuchungen zeigen, dass mit der Schnell- und Reaktivkraftleistung assoziierte Strukturen (z. B. Sehnen) bereits nach 2 Wochen mit negativen Adaptionen auf fehlende Trainingsreize reagieren, woraus eine herabgesetzte Leistungsfähigkeit resultieren kann [3]. Hierfür spricht ebenfalls eine erhöhte Inzidenz von Verletzungen der Achillessehne in Folge von Saisonpausen oder trainingsfreien Perioden [14]. Die Ergebnisse von Cohen et al. (2020) weisen darauf hin, dass auch hinsichtlich der intermuskulären Koordination die fehlenden fußballspezifischen Belastungen zu Veränderungen der Testleistung geführt haben könnten [7].

Einzig in der Untersuchung von Pedersen et al. [16] haben die Fußballspielerinnen ihre Sprint- bzw. Sprungleistung über den Zeitraum des Lockdowns aufrechterhalten [16]. Das Training während des Lockdowns beinhaltete jedoch, im Vergleich zu den weiteren eingeschlossenen Studienprotokollen, einen erhöhten Umfang an Schnelligkeits- und Sprungtrainingseinheiten. Die weniger strengen staatlichen Auflagen in Norwegen ermöglichten den in die Studie von Pedersen und Kollegen eingeschlossen Fußballspielerinnen schon früh Einzeltraining und Kleingruppentraining im Freien.

Diese Tendenzen werden von Untersuchungen gestützt, welche die Spielleistung männlicher Fußballspieler in kroatischen, polnischen und spanischen Profiligen vor und nach der Lockdownperiode verglichen. Hierbei zeigte sich, dass die Spieler vor allem hinsichtlich hochintensiver Aktionen (Sprints, Beschleunigungen) ein vermindertes Leistungsniveau aufwiesen [9, 19, 21]. Die Häufigkeit und Anzahl von Läufen mit niedriger und moderater Geschwindigkeit scheinen infolge der Lockdownperiode hingegen nicht negativ beeinflusst worden zu sein.

Die vorhandene Evidenz zeigt, dass Phasen mit reduzierten Belastungsumfängen und -intensitäten zu einer Reduktion der Leistungsfähigkeit des kardiovaskulären, neuronalen sowie muskuloskeletalen Systems führen [4]. Derartige Detraining-Anpassungen scheinen dabei in Abhängigkeit des betroffenen Systems in unterschiedlichem Maße und ungleicher Geschwindigkeit einzutreten [4, 5, 13]. Auch innerhalb der eingeschlossenen Studien zeigten sich bei differenzierter Betrachtungsweise Unterschiede in der Ausprägung und Richtung der Effekte des substituierten heimbasierten Trainings auf die konditionellen Fähigkeiten der Fußballspielenden in Folge des Lockdowns.

In Anbetracht der beschriebenen Befundlage sollte bei der Rückkehr professioneller Fußballspielenden in den regulären Trainings- und Wettkampfbetrieb nach einem Lockdown-bedingten Ersatztraining auf eine progressive und individuelle Belastungssteuerung insbesondere im Bereich des Schnelligkeits‑/Krafttrainings geachtet werden [14].

### Limitationen

Es liegen nur wenige Studien zu den Auswirkungen des COVID-19-Lockdowns auf die Leistungsfähigkeit von professionellen Fußballspielenden vor. Gleichzeitig weisen die eingeschlossenen Untersuchungen, u. a. bedingt durch die unterschiedlichen COVID-19-Maßnahmen der Länder, relativ große Differenzen in Bezug auf Dauer und Art der durchgeführten Ersatztrainings auf. Weiterhin unterschieden sich auch die Erhebungsmethoden sowie die erfassten Leistungsparameter der Studien in weiten Teilen, was die Aussagekraft der Ergebnisse einschränkt. Aufgrund des narrativen Charakters der vorliegenden Arbeit wurde die mögliche Einflussnahme der methodischen Qualität (inkl. Bias-Risiko) der eingeschlossen Publikationen auf die Ergebnisse nicht bewertet.

## Fazit für die Praxis

- In Folge des COVID-19-Lockdowns kam es im Frühjahr 2020 zum Erliegen des Wettkampfsports und starken Einschränkungen der Trainingsmöglichkeiten im professionellen Fußball.
- Phasen ohne sportartspezifische Belastung können in einer herabgesetzten Leistungsfähigkeit resultieren. Um derartigen Detraining-Anpassungen entgegenzuwirken, wurden heimbasierte Ersatztrainings mit unspezifischen Kraft- und Ausdauermethoden durchgeführt.
- Allgemeine Ausdauer- und Kraftfähigkeiten scheinen durch unspezifische Heimtrainingsprogramme adäquat erhalten werden zu können.
- Die häusliche Quarantäne schränkte dagegen vor allem das Schnelligkeits- und Schnellkrafttraining stark ein, was sich in einer herabgesetzten Sprint- und Sprungleistung widerspiegelte.
- Es scheint daher sinnvoll, die professionellen Fußballspielenden bei Wiederaufnahme des Trainings- und Spielbetriebes mittels progressiver und individueller Belastungssteuerung wieder an Läufe und Bewegungen mit maximaler Geschwindigkeit heranzuführen, um dem Auftreten von Verletzungen vorzubeugen.
